# Parental stress and the onset and course of childhood asthma

**DOI:** 10.1186/s13030-015-0034-4

**Published:** 2015-03-05

**Authors:** Noriko Yamamoto, Jun Nagano

**Affiliations:** Faculty of Arts and Science, Kyushu University, 6-1 Kasuga Park, Kasuga, Fukuoka 816-8580 Japan

**Keywords:** Psychological stress, Personality, Caregiver, Parent, Childhood asthma, Prospective study

## Abstract

The influence of a caregiver’s stress on the development of childhood asthma is an important aspect of the treatment and prevention of illness. Many cross-sectional studies have investigated the association between parenting attitude and/or caregiver’s stress and childhood asthma morbidity, but prospective studies are more advantageous than cross-sectional studies in interpreting a causal relationship from the results. We here present an overview of prospective studies that have reported a relationship between parental stress and the morbidity or course of childhood asthma and discuss the role of parental mental health in its prevention and treatment. Almost all of the studies referred to in this paper show that caregiver (mostly mothers) stress contributed to the onset and to a poor prognosis, while only a few studies have examined the adverse effect of paternal stress on childhood asthma. Their results are inconsistent, and there is insufficient data examining specific stress-related properties that can be targeted in intervention studies. Not only maternal but also paternal influence should be considered in future studies, and it will be important to assess specific stress-related properties that can be the foundation of specific intervention methods.

## Introduction

In the past it was thought that asthma occurred more frequently in populations from developed countries, but there has been a sharp increase in the prevalence of asthma in some developing countries, making it a major worldwide public health problem [1]. The overall world burden of asthma is increasing, particularly that of children [2]. The International Study of Asthma and Allergies in Childhood (ISAAC) [3], the most comprehensive survey of asthma, rhinoconjunctivitis, and eczema of children, provides information about the prevalence and severity of these diseases. Globally, the prevalence of asthma in the 6-7-year age group was 11.7%, while in the 13-14-age group the prevalence of current asthma was 14.1%. It was reported that intermediate prevalence values for asthma are found in the Asia-Pacific region, including Japan, in the 6-7-age group, while low prevalence values for asthma were found in the 13-14-age group. In the younger age group there were more boys than girls with asthma. In the older age group there were more girls than boys with asthma. In both, socioeconomic status was significantly correlated with current symptoms of asthma.

**Table 1:** **Prospective studies reporting an association between caregiver stress and childhood asthma**

**Author, year**	**Caregiver**			**Children**				**F**/**U Period**	**Results**
	**Attribute**	**Stress and relevant factors**		**Attribute**	**N**	**Age, baseline**	**Outcomes**		
Onset of disease									
Wright, 2002	Caregivers	Perceived stress		Predisposed	496	2 to 3 m	Wheezing	1y	Positive assoc. b/w caretakers’ perceived stress and child's wheezing occurrence
Milam, 2008	Parent	Perceived stress		Healthy	2888	5 to 6 y	Wheezing	1y	Positive assoc. b/w parental stress and child's wheezing incidence, in parents with asthma or in boys
Wood, 2011	Mother	Pregnancy anxiety, postnatal depression, perceived stress, difficult life circumstances		Predisposed	560	Pre-born	Wheezing	1y (2nd/3rd trimester to age 1y)	Positive assoc. b/w mother's stress/depression and multiple wheezing
Reyes, 2011	Mother	Prenatal/postnatal demoralization		African Americans/Hispanics	279	Pre-born	Wheezing	5y (3rd trimester to age 5y)	Positive assoc. b/w mother's prenatal demoralization with wheezing
Guxens, 2014	Mother and Father	Psychological distress, depression, anxiety		General population	4848	Pre-born	Wheezing, age 1 to 4y; asthma diagnosis, age 6y	6y (2nd trimester to age 6y)	Positive assoc. b/w mother's prenatal distress/depression/anxiety and child's wheezing/asthma
Course of disease									
Weil, 1999	Parent	Mental health, life stress, social support, parenting style		Patients with asthma	1528	4 to 9 y	Asthma symptoms, wheezing, hospitalization	9 m	Positive assoc. b/w mental health and hospitalization
Bartlett, 2004	Mother	Depression		Patients with asthma	158	8y in average	Asthma morbidity, ED visits	6 m	No assoc. b/w mother's depression and morbidity; Some assoc. b/w mother's depression and a constellation of beliefs and attitudes that may influence adherence
Nagano, 2010	Mother	Behavioral pattern associated with chronic stress, parenting style		Patients with asthma	223	2 to 12 y	Asthma symptoms (severity)	1y	<7y: positive assoc. b/w chronic irritation/emotional suppression and asthma severity; no assoc. with parenting style. 7+ y: positive assoc. b/w over interference and asthma severity; negative assoc. with egocentric behavior.
Lange, 2011	Mother and Father	Fathers: PTSD, depression, antisocial behaviour Mothers: depression		Puerto Rican twins and their parents	339 pairs	1 y	Asthma symptoms, oral steroid use, hospitalization	2y	Positive assoc. b/w paternal depression and oral steroid use, maternal depression and hospitalization

In Japan, the 2013 school health statistical survey, conducted yearly by the Ministry of Education, Culture, Sports, Science and Technology, reported that the prevalence of childhood asthma was 2.13% at the age of 5 (kindergarten), 4.15% in the overall elementary school age group, and 3.22% in the overall junior high school age group. The highest prevalence values for asthma were found at the age of 6, then they decreased gradually with age. In all age groups, the prevalence of asthma was slightly higher for boys than for girls. Since the Japan Society of Pediatric Allergy and Clinical Immunology published the Japanese Pediatric Guidelines for the Treatment and Management of Asthma (JPGL) in 2002, inhaled corticosteroids have become widely used, decreasing the number of deaths and severe cases, but this trend has leveled off in the past few years.

The risk factors of childhood asthma include a predisposition toward allergies, airway hyperreactivity, sex (as a biological factor), inhalant allergens, food allergens, respiratory infections (such as viruses), air pollution, passive smoke inhalation, exercise, drugs, emotion and stress, including psychological, physical, and socioeconomic stress (as environmental factors). Psychological stress due to the relationship with caregivers is unavoidable for children, and it has a significant impact on the onset and course of childhood asthma.

Clinicians know that parents in a stressful situation or who have specific parenting attitudes can become a stressor that affects the disease status of their child’s asthma. Many studies have reported positive associations between psychological stress suffered by parents, or ‘parental stress’, and childhood asthma morbidity. There are many cross-sectional studies that have investigated the influence of parenting attitudes and stress on childhood asthma morbidity [4,5]. Determining causality from the results of retrospective study is rather difficult. Prospective studies are better than cross-sectional studies in that a causal relationship can be shown from the results. For example, Wright et al. reported in a prospective study that followed asthmatic infants aged 2–14 months that while parental stress predicted a child’s wheezing, a child’s wheezing did not affect parental stress [6]. For these reasons, we here present an overview of prospective studies that have reported a relationship between parental stress and the morbidity or course of childhood asthma and discuss the role of parental mental health in the prevention and treatment of childhood asthma.

## Overview of prospective studies

PubMed was searched using terms related to ‘childhood asthma’, ‘parental stress’ or ‘parental mental health’, and ‘prospective study’.

### Parental stress and the onset of childhood asthma

Several studies have prospectively investigated the association between maternal stress and childhood asthma, with many targeting children who were genetically predisposed to asthma (Table 1). Wright et al. [6] conducted a prospective birth-cohort study that investigated the influence of the perceived stress of caregivers (96.6% mothers) on the wheezing of infants who were genetically predisposed to asthma. Infants and their family members with a history of asthma or allergy were recruited. At an initial home visit, when each child was two to three months of age, data were collected on home environmental exposure and caregiver stress through personal-interview questionnaires. Subsequently, six bimonthly telephone questionnaires ascertained changes in caregiver stress and the occurrence of respiratory illness in the children. Greater levels of caregiver-perceived stress at baseline was associated with increased risk of subsequent repeated wheezing by the children. Caregiver-perceived stress remained significant after controlling for parental asthma, socioeconomic status, birth weight, and race/ethnicity. In the Urban Environment and Childhood Asthma study, a prospective, observational birth cohort study of 560 children with at least one parent who had allergy or asthma, Wood et al. investigated the association between maternal stress and the wheezing of the child [7]. The enrolled mothers underwent a prenatal study visit (in the 2nd/3rd trimester of pregnancy) and stress-related questionnaires were administered that contained questions about maternal anxiety during pregnancy, perceived stress, and difficult life circumstances. Cord blood cell samples were collected at the time of the child’s birth to assess mononuclear cell cytokine responses. Postnatal depression of mothers was determined by self-administered questionnaires, and postnatal telephone surveys were administered every three months to assess the child’s respiratory and allergy symptoms. In addition, at the age of three months the study staff visited the child’s home to administer a home environment questionnaire and to collect household dust samples. Finally, all infants were seen at 12 months of age by a study physician in the research clinic, at which time a physical examination was performed and blood samples were collected to assess sensitization to common allergens (allergen-specific IgE). Positive associations were detected between multiple bouts of wheezing and maternal stress and maternal depression, after adjustment for environmental factors (dust allergen, endotoxin, indoor nicotine, and dioxide level).

In another study the subject of the investigation was a population at high risk for asthma. Reyes et al. examined the relation of maternal demoralization with wheezing and seroatopy among children living in a low-income urban community [8]. African American and Hispanic (mainly Dominican) women were recruited during pregnancy. Maternal demoralization (i.e., psychological distress) was measured both pre and postnatally. The outcome, their child’s wheezing, was determined on scheduled visits at 14 time points (every three months from birth to three years of age, then every six months up to five years of age). Prenatal demoralization significantly predicted overall wheezing, transient (birth to 2.5 years of age) wheezing, and persistent (birth to five years of age) wheezing.

In a population-based prospective cohort study not limited to children at high risk for asthma, Milam et al. [9] recruited healthy five-to six-year-old school children without asthma for the evaluation at study entry of parent-reported wheezing by the child and parent perceived stress. Wheezing by the child was evaluated one year later. No relation was found between parental stress and childhood wheezing after one year of follow-up for the entire cohort, but among boys with no parental history of asthma, stress was associated with wheezing in a dose-dependent manner. Guxens et al. performed a prospective population-based cohort study [10]. Maternal psychological distress (overall distress, depression, and anxiety) was assessed at the second trimester of gestation, at two and six months after delivery, and finally at three years after delivery. Paternal psychological distress was also assessed at the second trimester of gestation and at three years after delivery. Wheezing by the children was annually examined by questionnaire. Physician-diagnosed ever asthma was reported at six years. Maternal psychological distress during pregnancy was associated with increased odds of wheezing by a child at all the observed time points (during the first six years of life), while paternal distress during pregnancy and maternal and paternal distress after delivery were not associated with childhood wheezing.

### Parental stress and the course of childhood asthma

Previous studies have shown that behavioral and psychological factors affect the asthma morbidity of asthmatic children. Weil et al. prospectively examined the relation between psychological factors and asthma morbidity among inner-city children [11]. Children four to nine years of age with asthma living in the inner city and their primary caretakers (97% mothers) were recruited. Psychological variables of both the children and caretakers, including the child’s and caretaker’s mental health, caretaker’s problems with alcohol, life stress, social support, and parenting style, were assessed at baseline. Asthma morbidity was evaluated at baseline and at three, six, and nine-month follow-up intervals. Caretaker and child mental health were important predictors of subsequent asthma morbidity, although they were related to different aspects of morbidity. Children whose caretakers had clinically significant mental health problems were hospitalized for asthma at almost twice the rate of children whose caretakers did not have significant mental health problems. Children with clinically significant behavior problems had significantly more days of wheezing and poorer functional status. Bartlett et al. examined the relation between maternal depressive symptoms and subsequent asthma morbidity of the child [12]. Elementary school-aged children (i.e., kindergarten through 5th grade, average eight years-old) with an asthma diagnosis on their school health records and their mothers were recruited. The study population was young minority children with asthma living in the inner city, with 98% of the families African American. Maternal depressive symptoms were not associated with child asthma morbidity, but were associated with a constellation of beliefs and attitudes that might significantly influence adherence to asthma medications and illness management.

One prospective study showed that the association between a mother’s psychological properties and her child’s asthma is considerably different according to the child’s age. Nagano et al. examined the relations between a mother’s stress-related conditions and parenting attitude and their child’s asthmatic status [13]. Mothers of an asthmatic child who visited an out-patient clinic were enrolled and completed a questionnaire that included questions about their stress/coping behaviors, parenting attitudes, and their child’s disease status. One year later, the child’s disease status was assessed by a mailed questionnaire completed by the mother, and the association between each type of parental stress/coping behavior and parenting style and the child’s asthmatic status was analyzed. The associations were different for children aged under seven years and for children aged seven years and over. Chronic irritation/anger and emotional suppression were aggravating factors for children aged under seven years, while the mother’s egocentric behavior was a mitigating factor and interference was an aggravating factor for children aged seven years and over.

In a cohort of young Puerto Rican twins, Lange et al. analyzed the relation between parental psychological stress, of both mothers and fathers, and the asthma morbidity of twins [14]. Parents of 339 pairs of twins were questioned about psychological stress and about the asthma of their children within the first year of life. The parents were again interviewed about their child’s asthma at age three. Paternal PTSD symptoms, depression, and antisocial behavior were each associated with increased asthma at age one. Maternal depressive symptoms were associated with an increased risk of asthma hospitalization at age one. At age three, maternal depressive symptoms were associated with an asthma diagnosis and hospitalization for asthma. In an analysis combining one and three-year outcomes, paternal depression was associated with repeated oral steroid use, and maternal depressive symptoms were associated with repeated asthma hospitalizations and asthma diagnosis. This is the first report of an independent association between indicators of paternal psychosocial stress and asthma outcomes in early childhood.

This summary of prospective studies shows that it is highly possible that parental stress has a significant influence on the onset and course of their child’s asthma. Studies have consistently shown that a caregivers’ stress contributes to the onset [6-9] and to a poor prognosis [11-15]. Below we discuss mechanisms and possibilities for future studies.

### Mechanism

When we consider the possible mechanisms by which parental stress affects childhood asthma, the first consideration should be the ways in which the parent has attempted to promote their child’s physical development and health. With the onset of childhood asthma, improvement of health management by the parents might include efforts to reduce allergen exposure, which could be counterproductive because improvements of hygiene due to the modernization of the living environment have been shown to lead to an increase in allergic diseases. Altered colonization with pathogenic and non-pathogenic bacteria during infancy has been linked to an elevated risk of allergy [16]. This makes it difficult to associate parenting styles with the development of allergic diseases. When parents are in a stressful situation, they may reduce support for their children during the course of asthma treatment. However, in regards to antigen depletion and medical treatment, it is difficult to show how parental support, such as attention to adherence, can underlie a major mechanism that is related to the onset and course of the illness. For example, Bartlett and colleagues reported that mothers with depressive symptoms at baseline had significantly more problems with adherence, such as the proper use of inhalers at six months, while medication adherence was not predictive of subsequent asthma morbidity or emergency department use [12].

Several studies have reported mechanisms underlying the exacerbation of asthma via a psycho-physiological pathway. Specific emotional states may influence the autonomic reactivity and pulmonary function of asthmatic children. Research shows that while sadness induces cholinergic airway contraction, happiness causes airway relaxation [17] and that cortisol response to psychosocial stress is blunted in asthmatic children [18]. Ippoliti et al. found that psychological stress can decrease response to sublingual immunotherapy (SLIT) in stressed asthmatic children [19]. Weil and colleagues reported that children with clinically significant behavior problems had significantly more days of wheezing and poorer functional status [11].

If the psychological stress of children in fact influences the course of the illness via a psycho-physiological pathway, we must consider the influence of parental or caregiver behavior on the child’s stress. Understanding the parent–child relationship and parenting styles may give us clues to the process. Mrazek et al. reported, in an inception cohort study of children born to asthmatic mothers, that parenting difficulties of mothers at three weeks of age was significantly associated with the early onset of asthma during a follow-up period of three years [20]. In this study, parenting difficulties when the infant was three weeks old were also associated with asthma persistence between ages six and eight [21]. Nagano et al. reported that maternal interference predicted poorer disease status one year later for relatively older children, aged seven to twelve years [13]. This association was not found for younger children (aged under seven years). A study by Weil also found no association between parenting style and poor prognosis of asthma for children aged four to nine years. There is insufficient data to derive concrete conclusions because the parent–child relationship allows multiple interpretations and because these associations between parenting styles and asthma morbidity may change as children grow.

How does parental behavior, in a more general sense and without being limited to the parent–child relationship or parenting style, influence a child’s asthma via the child’s psychological stress? It has been consistently shown that parents in an unfavorable mental state, such as "depression", "anxiety", "stress", or "chronic irritation", may predict a poorer status for a child’s asthma. Wolf et al. prospectively examined the association between parent psychological characteristics and longitudinal changes in the inflammatory markers of children with asthma [15]. Fifty children with asthma (9 to 18 years old, average 13.3 years) and 33 healthy children (average 13.5 years) were assessed for eosinophil cationic protein (ECP) and stimulated interleukin-4 (IL-4) production as asthma-relevant inflammatory markers at baseline and at follow-up an average of 208 days later. Parent depression and perceived stress were assessed at baseline and child depression and anxiety at follow-up. Higher levels of parental perceived stress at baseline were associated with a greater increase over time in the child’s ECP release and IL-4 production, and higher levels of parental depression at baseline were associated with an increase in ECP over time, both for children with asthma and healthy children. These changes in inflammatory markers suggest that a parent’s perceived stress or depression may be transmitted verbally or nonverbally to their child and may cause biological allergic responses by the child.

Interestingly, several studies have suggested that stress-elicited disruption of immunity may already begin in utero. Wright et al. prospectively examined associations among prenatal maternal stress and cord blood mononuclear cell (CBMC) cytokine responses in a high-risk, inner city, ethnic minority population [22]. The 557 mothers recruited during the 3rd trimester of pregnancy (gestational age 34 weeks or more) were assessed for prenatal maternal stress. Prenatal maternal stress included financial hardship, difficult life circumstances, community violence, and neighborhood/block and housing conditions. The majority of the mothers were from ethnic minorities (Black; 71%, Latino; 19%) living in an area with more than 20% of the residents below the poverty level. The mother or father of the child had a history of allergic diseases. CBMCs were stimulated by various innate and adaptive stimuli, and cytokines were measured using multiplex ELISAs. Higher prenatal stress was related to increased IL-8 production after microbial (cytosine-phosphate-guanine dinucleotides, polyinosinic-polycytidylic acid, peptidoglycan) stimuli and increased TNF-alpha to microbial stimuli (cytosine-phosphate-guanine dinucleotides, polyinosinic-polycytidylic acid). In adaptive immune responses, higher stress was associated with increased IL-13 after dust mite stimulation and reduced phytohemagglutinin-induced IFN-gamma. The results suggest that there is an interaction between maternal, placental, and fetal factors that may underlie aspects of the programming of fetal development. Maternal stress has been associated with altered fetal programming of the developing hypothalamic-pituitary axis (HPA) and nervous system through increased maternal glucocorticoids, pro-inflammatory cytokines, altered nutrient availability and dysregulated leptin and insulin signaling that are transmitted to the fetal compartment via placenta [23]. If this is true, children born to a stressed mother may already be predisposed to allergic diseases at birth, and stress-induced perinatal immunomodulation may contribute to an increasing risk of asthma [7,8,10].

An epigenetic mechanism has also been implicated as a potential factor linking psychological stress to childhood asthma. Ian C.G.Weaver et al. reported that the adult offspring of mother rats that naturally show an increased frequency of pup licking/grooming and arched back nursing show decreased methylation of the exon 1Z consensus sequence of the glucocorticoid receptor gene [24]. This finding suggests that maternal care of pups influences the development of HPA responses to stress through an epigenetic mechanism. Chen and colleagues found that increased methylation of a single CpG in the ADCYAP1R1 promoter is associated with an increased risk of asthma for Puerto Rican children, particularly for children exposed to violence [25,26]. ADCYAP1R1 encodes a membrane receptor for pituitary adenylate cyclase-activating peptide and is highly expressed in the hypothalamus and limbic structures that are integral to stress response. This finding suggests that exposure to trauma in childhood specially changed ADCYAP1R1 methylation in a way that, in turn, alters transcriptional regulation of this gene in a manner that increases asthma risk.

### Possibilities for future studies

As shown above, the influence of a caregiver’s stress on the development of childhood asthma is an important aspect of the treatment and prevention of illness. However, data obtained from these previous studies are insufficient to explain many of the problems that occur in family based intervention in the treatment of children with asthma. The previous studies have limitations that must be addressed.

In almost all of the prospective studies overviewed here, the caregiver under examination was limited to the mother or the surveyed subjects were primary caregivers, most of whom were the mother. Few studies have examined the adverse effects of paternal stress on a child’s asthma. However, paternal depression may contribute to the exacerbation of a child’s asthma through discord between the parents or through negative maternal parenting behaviors, and some fathers have a stronger effect than mothers on their child’s disease status as the child grows. Schöbinger et al. investigated paternal critical attitude and negative father-child communication in families with an asthmatic child. Twenty-seven children with asthma (six to thirteen years old) and twenty-three healthy children and their fathers participated in the study. Paternal critical attitude and negative father-child communications were assessed by analysis of a videotape recording [5]. Significantly more fathers of the asthmatic children than of the controls showed a critical attitude, and more long sequences of negative verbal communication occurred in father-child dyads with asthma than in the control dyads. No significant relationship was found between either the father’s critical attitudes or the amount of negative verbal communication and the severity of the child’s asthma, compliance, or serum IgE status. Lim et al. tested a hypothesized model of the relationship among parental depressive symptoms, parenting behaviors, and a child’s asthma disease activity [27]. In this study, 106 children with asthma (seven to seventeen years old) and their parents participated. Maternal depressive symptoms appeared to affect the child’s asthma morbidity directly by contributing to the mother’s negative parenting behaviors. Paternal depressive symptoms, weaker than maternal depressive symptoms in relation to their child’s asthma, appeared to contribute to greater interparental negativity, which contributed to maternal negative parenting, which in turn, indirectly contributed to the child’s asthma morbidity. The discrepancy in the effect of maternal versus paternal negative parenting is consistent with previous meta-analytic findings conducted by Connell and Goodman [28]. However, because these studies are retrospective care must be taken in interpreting the results. In fact, the results of the studies referred to above are not the same. Lange et al. reported an adverse effect of paternal stress on a child’s asthma morbidity [14], while no significant association between paternal stress and the onset of childhood asthma was found by Guxens et al. [10]. Further prospective studies will be necessary to clarify the discrepancy.

In many studies, caregiver “stress” is assessed by measuring non-specific parameters such as “perceived stress”, “depression”, and “anxiety”. However, the cause of falling into a negative mental state may differ significantly from one individual to another, and the “severity” of a mental disorder may also differ depending on the cause, resulting in differences in the effect on the child’s disease status. In order to properly interpret the causal relation between a caregiver’s stress and a child’s asthma, even prospective studies lack the necessary evidence. Therefore, it will be important to perform intervention studies that target stress to prove that the mitigation of stress can lead to the improvement of a child’s asthma or to a reduced risk of asthma. In such intervention studies, non-specific parameters such as perceived stress, depression, and anxiety cannot be used. Environmental factors, including socioeconomic factors and life environment, and the behavioral characteristics of each caregiver, especially stress/coping behaviors, underlie the problems of caregivers. It is difficult for clinicians to manage the former environmental factors, but it is possible to address the individual psychological factors. With this point in mind, Nagano et al. focused on specific behavioral characteristics (stress/coping behaviors) of mothers [13]. The results suggest that some types of maternal stress/coping behaviors had great impact on a child’s asthma morbidity one year later; maternal chronic irritation/anger related to an interpersonal relationship, emotional suppression, and an altruistic tendency of mothers were aggravating factors for children aged under seven years, while for children aged seven and over the mother’s egocentric behavior was a mitigating factor. Therefore, intervention using psychotherapy that targets individual properties may be effective in planning family based strategies.

## Conclusions

The influence of a caregiver’s stress on the development of childhood asthma and on disease status indicates possible targets for treatment and prevention. In order to plan practical treatment strategies, not only maternal but also paternal influence should be considered in future studies. It will be important to assess stress-related properties that are potential targets of effective intervention methods. Clarifying and interpreting causal relationships through intervention studies then confirming the effect of the causality will be necessary.
